# Ocular Ultrasonography for Acute Ischemic Stroke: A Focus on Optic Nerve Sheath Diameter and Ischemia Side Correlation

**DOI:** 10.7759/cureus.93574

**Published:** 2025-09-30

**Authors:** Hande Asan, Sehmuz Zengin, Ozlem Dikme, Ozgur Dikme, Ekin Dumanli, Hakan Topaçoğlu, Erdem Cevik

**Affiliations:** 1 Emergency Medicine, Sultan 2. Abdulhamid Han Training and Research Hospital, Istanbul, TUR; 2 Emergency Medicine, Diyarbakır Gazi Yasargil Training and Research Hospital, Diyarbakır, TUR; 3 Emergency Medicine, Istanbul Training and Research Hospital, Istanbul, TUR; 4 Emergency Medicine, Turkish Hospital, Doha, QAT; 5 Emergency Medıcıne, Dr. Suat Günsel University of Kyrenia Hospital, Kyrenia, CYP

**Keywords:** acute ischemic stroke, cerebrovascular accident, emergency department, optic nerve sheath diameter, ultrasonography

## Abstract

Background: Ultrasound devices are nowadays widely available in emergency departments (ED). In recent years, ocular ultrasonography (OUS) has become a reliable non-invasive method for detecting an enlarged optic nerve sheath diameter (ONSD), serving as a valuable indicator of elevated intracranial pressure (ICP). In stroke patients, ICP may also increase depending on the severity of the condition. This study aimed to evaluate the relationship between the side of the ischemic lesion and the ONSD using bedside OUS in patients with acute ischemic stroke.

Materials and methods: This prospective single-center cross-sectional study was conducted between July and September 2016. All patients aged 18 years and older who presented to the ED and were diagnosed with acute ischemic stroke based on diffusion-weighted imaging-magnetic resonance imaging (DWI-MRI) findings were enrolled. The ONSD was measured at 3 mm behind the globe using a 10 MHz linear transducer on the closed eyelids. Demographic data, neurologic deficits, duration of symptoms, National Institutes of Health Stroke Scale (NIHSS) score, ischemia side, and ONSD for both eyes were recorded.

Results: Sixty-seven patients were enrolled in the study. The mean±SD ONSD was observed as 4.85±0.90 mm for the right and 4.86±1.01 mm for the left. No statistically significant difference was found between the right and left ONSD values in patients with right-hemispheric stroke. This was similarly observed in patients with left-hemispheric stroke. There was no statistically significant difference between age, gender, duration of symptoms, and NIHSS score.

Conclusion: In patients with acute ischemic stroke, measuring one eye is sufficient for ONSD assessment. There is no correspondence between the ischemia side and ONSD values.

## Introduction

Ischemic stroke is the second most common cause of death and a major cause of disability in the world [1]. Early deterioration of mental status and death are often results of edema in the infarcted tissue [2]. Increased intracranial pressure (ICP) resulting from cerebral edema may develop due to impaired cerebral autoregulation. Although deterioration in neurological examination is an early indicator of rising ICP, its assessment can be challenging in sedated patients or those with altered mental status [3]. Traditionally, ICP has been monitored through invasive techniques involving intracranial catheter placement or via imaging modalities such as computed tomography (CT) and magnetic resonance imaging (MRI), which require patient transport and may involve exposure to ionizing radiation [4].

Numerous studies have investigated how the optic nerve may indirectly reflect ICP through its relationship with the subarachnoid space. These studies have identified that increased cerebrospinal fluid (CSF) pressure in the subarachnoid space leads to edema in the optic nerve sheath, caused by the movement of CSF as ICP rises [3,5-8]. Recently, ocular ultrasonography (OUS) for detecting a widened optic nerve sheath diameter (ONSD) has become a reliable, non-invasive marker of elevated ICP [9]. After a short training session, emergency physicians have proven that bedside OUS to measure ONSD can be effectively performed, offering valuable information about increased ICP in patients with ischemic stroke, head trauma, preeclampsia, and central nervous system infections [10]. Measuring ONSD using bedside OUS in the emergency department (ED) provides a quick, consistent, and non-invasive method to assess elevated ICP, especially in critically ill or hemodynamically unstable patients for whom transport and radiation exposure present significant risks.

While the utility of ONSD in assessing stroke severity, infarction site, and clinical outcomes has been addressed in previous studies, the relationship between ONSD and the side of ischemia remains underexplored [11-13]. Considering the well-established association between elevated ICP and ONSD, this study aims to investigate whether bedside ultrasonographic measurement of ONSD is associated with the side of ischemia in patients presenting with acute ischemic stroke.

## Materials and methods

Study design and setting

This prospective, cross-sectional study was conducted in the Emergency Medicine Department of Istanbul Training and Research Hospital, Istanbul, Turkey. Ethical approval was authorized by the Bakırköy Dr. Sadi Konuk Training and Research Hospital Clinical Research Ethics Committee (approval number: 2016-214). The study was conducted between July and September 2016 with patients presenting to the ED within 24 hours of stroke symptom onset. All consecutive patients over 18 years of age and who were diagnosed with acute ischemic stroke by diffusion-weighted imaging-magnetic resonance imaging (DWI-MRI) and provided informed consent to participate in the study were included. The exclusion criteria were as follows: lack of written or verbal consent and presence of other conditions associated with ONSD widening (optic neuritis, optic nerve arachnoid cysts, optic nerve injury, intracranial mass, pseudotumor cerebri, cerebral venous sinus thrombosis, intraocular inflammation, subarachnoid hemorrhage, anatomical eye defect, glaucoma, and multiple sclerosis). Age, sex, neurologic deficits, duration of symptoms, National Institutes of Health Stroke Scale (NIHSS) score, ischemic lesion side, and ONSD measurements were noted. Written informed consent was obtained from each patient or their relative.

Measurements

OUS was performed using a General Electric Logiq 9 ultrasound machine (GE HealthCare, Chicago, IL, USA) with a 5-10 MHz linear probe. Measurements were obtained with the patient in the supine position, using the closed eyelid technique. Both eyes were scanned in the sagittal plane, and ONSD was measured 3 mm posterior to the point where the optic nerve enters the globe. The sonographic diameters of the left and right ONSD were recorded without causing any delay in the diagnostic and treatment process. Two measurements were taken for each eye, and the mean of these measurements was calculated. The sonographic procedure was carried out in the ED by two emergency physicians who had undergone training in OUS and had four years of clinical experience with sonography, including over 200 ocular ultrasound examinations. The ultrasound examiners were blinded to the symptoms, neurological examinations, and neuroimaging findings and ischemic side.

Outcomes

The primary outcome of this study was to compare ONSD measurements between the right and left eyes in patients with acute ischemic stroke using bedside OUS. Secondary outcomes included evaluating the relationships between ONSD values and NIHSS scores, the side of cerebral ischemia, and patients' demographic characteristics.

Statistical analyses

IBM SPSS Statistics for Windows, V. 15.0 (SPSS Inc., Chicago, IL, USA), was used. The number and percentage for categorical variables, mean, standard deviation, and minimum-maximum for numerical variables were used for descriptive statistics. Two group comparisons were used for the t-test, as the numerical variables for the intergroup comparisons provided the normal distribution condition. Differences between groups of categorical variables were tested by chi-squared analysis. Correlation between independent continuous variables was assessed by Pearson correlation. A statistical significance level of alpha was accepted as a p-value less than 0.05. The sample size was calculated to be 67, with a power of 80% and an alpha error of 0.05.

## Results

In this prospective study, 67 patients were evaluated, of whom 37 (55.2%) were male. The mean age of all patients was 68.4 (SD±13.1) years. The median symptom onset time was 14 hours. When the vascular territories were examined, the most common ischemic lesion was observed in the middle cerebral artery (MCA) territory (n=37; 55.2%) (Table 1). There was no statistically significant difference in the distribution of vascular territories between patients with right- and left-hemispheric ischemic lesions (p=0.457). Thirty-five (52.2%) patients had a right hemisphere infarct, and 32 (47.8%) patients had a left hemisphere infarct. No statistically significant differences were found between the right and left infarct groups regarding sex, age, and symptom onset time (Table 2).

**Table 1:** Distribution of vascular territories causing infarction DWI-MRI: diffusion-weighted imaging-magnetic resonance imaging; MCA: middle cerebral artery; ACA:anterior cerebral artery; PCA: posterior cerebral artery

		DWI-MRI			
		Right ischemic lesion		Left ischemic lesion	
		n	%	n	%
Vascular territories	MCA	18	51.4	19	59.4
	ACA	1	2.9	3	9.4
	PCA	7	20.0	6	18.8
	Posterior circulation	9	25.7	4	12.5

**Table 2:** Analysis of demographic data according to ischemia side ^a^Chi-squared test.^ b^Independent samples t-test DWI-MRI: diffusion-weighted imaging-magnetic resonance imaging; IQR: interquartile range

		DWI-MRI				p
		Right ischemic lesions		Left ischemic lesions		
		n	%	n	%	
Sex^a^	Male	19	54.28	18	56.25	0.872
	Female	16	45.72	14	43.75	
	t	Mean±SD	Median (IQR)	Mean±SD	Median (IQR)	p
Age^b^	-0.176	68.13±11.92	67 (58-80)	68.71±14.52	72 (62.88-76)	0.861
Symptom onset time (hour)^b^	0.588	22±21.91	15 (11-26)	19.33±15.70	13 (8-30)	0.584

When the symptoms were examined, dysarthria was the most frequent (58.2%). Patients with left-sided ischemic lesions had a statistically significant difference in dysarthria compared to those with the right (p=0.008), and no statistically significant difference was found in the other symptoms. The mean NIHSS score was calculated as 3.55±3.75 points (interval 0-19). There was no statistically significant difference between mean ONSD measurements and NIHSS scores in both eyes (p=0.94).

The mean ONSD values for the right and left eyes are presented in Table 3. There was no statistically significant difference between the mean ONSD on the ischemic side and that on the non-ischemic side. The mean ONSD values in both eyes of patients with left-sided infarcts were higher than those in patients with right-sided infarcts; however, the differences were not statistically significant.

**Table 3:** Comparison of right and left ONSD in patients with right- and left-hemispheric ischemic stroke lesion ^a^Paired samples t-test. ^b^Independent samples t-test ONSD: optic nerve sheath diameter

	Mean right ONSD (mm)±SD	95% CI (mm)	Mean left ONSD (mm)±SD	95% CI (mm)	t^a^-p
Right ischemic lesion	4.71±0.87	4.34-4.99	4.75±0.98	4.42-5.09	-0.957-0.649
Left ischemic lesion	5±0.93	4.56-5.35	4.97±1.05	4.59-5.35	0.268-0.810
t^b^-p	-1.18-0.203		-0.878-0.385		

**Figure 1:**
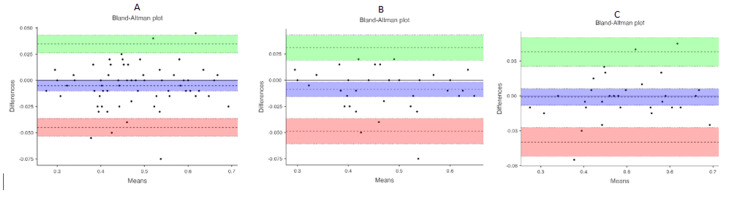
Bland-Altman analyses for the mean right and left optic nerve sheath diameters (A) All enrolled participants. (B) Participants with right ischemic stroke lesions. (C) Participants with left ischemic stroke lesions.

ONSD measurements on the ischemic and non-ischemic sides were strongly and positively correlated (right-sided infarcts: Rho=0.797 and p<0.001; left-sided infarcts: Rho=0.742 and p<0.001). No significant correlation was found with age, duration of symptoms, or NIHSS scores (Table 4). The Bland-Altman analysis demonstrated minimal bias (−0.00507) between right and left eye ONSD measurements in all enrolled participants, with narrow 95% limits of agreement (−0.04492 to 0.03477), indicating good agreement and potential interchangeability between the two methods. Separate analyses of patients with left- and right-hemispheric ischemic stroke lesions also demonstrated minimal bias and acceptable limits of agreement between right and left eye ONSD measurements, supporting the interchangeability of bilateral ONSD assessments in these populations (Figure 1).

**Table 4:** Correlation between ONSD and NIHSS score, duration of symptoms, and age Pearson correlation test. ONSD: optic nerve sheath diameter; NIHSS: National Institutes of Health Stroke Scale

	Mean right ONSD		Mean left ONSD
Right ischemic lesion	Rho-p	Left ischemic lesion	Rho-p
Mean left ONSD	0.797-<0.001	Mean right ONSD	0.742-<0.001
Age	-0.035-0.844	Age	-0.241-0.184
Symptom onset time	0.115-0.511	Duration of symptoms	0.099-0.591
NIHSS score	0.225-0.194	NIHSS score	-0.287-0.111

## Discussion

Correlation analyses showed a significant association between right and left mean ONSD measurements; however, no significant correlations were observed between ONSD and NIHSS score, patient age, or symptom onset time. Similarly, Çömez et al. reported a strong positive correlation between right and left ONSD values in patients with ischemic stroke, while no correlation was found between ONSD and sex, age, NIHSS score, or symptom duration [11]. Conversely, in a group of patients receiving thrombolytic therapy, ONSD and NIHSS scores assessed before treatment and 24 hours afterward showed a positive correlation [14]. In this study, we measured each patient's NIHSS score and ONSD only at diagnosis. However, we believe that if NIHSS scores and ONSD values had been measured repeatedly at specific intervals in the same patients, a correlation might have emerged.

Yildiz et al. found that, among 82 patients with ischemic stroke, 48.8% were female and the mean age was 67.5 years [15]. In another study examining the vascular distribution in ischemic stroke, MCA infarction was the most common infarct type, occurring in 51% of cases [16]. The vascular territories causing infarction and patient characteristics in the present study were consistent with those reported in previous research.

Contrary to our hypothesis, our results did not demonstrate a statistically significant relationship between the ischemic side and ONSD. The mean ONSD measurements for the right and left eyes were similar, regardless of whether the patient had a right- or left-sided infarct. According to the literature, studies that included various clinical populations, including traumatic brain injury and ischemic or hemorrhagic stroke, have consistently reported no statistically significant differences between the right and left ONSD, indicating that measuring ONSD in one eye is sufficient [15,17,18]. However, no studies have directly compared ONSD values between the side of the ischemic lesion and the contralateral side. Furthermore, none have reported a mean difference between ipsilateral and contralateral ONSD measurements nor provided lesion lateralization data related to ONSD values. Therefore, in designing this study, we aimed to determine whether a relationship exists between the side of the ischemic lesion and the ONSD values measured in each eye in patients with acute ischemic stroke. Nonetheless, we found that, in patients with acute ischemic stroke, increases in ONSD were positively correlated and similar in both eyes. Anatomically, the optic nerve is an extension of the central nervous system and is surrounded by the arachnoid mater, dura mater, and CSF [5]. Likely due to this connection, brain edema and increased ICP caused by ischemic stroke reflect similarly throughout the entire cranial cavity, affecting both the right and left optic nerve sheaths independently of the side of ischemia.

Previous studies have reported normal ONSD values in healthy individuals ranging from approximately 3.7 mm to 5.1 mm, depending on the imaging modality and study population [19-21]. Although elevated ICP has been associated with ONSD values between 4.8 mm and 5.9 mm, no universally accepted pathological threshold has been established [6,8,9,22]. In studies evaluating ischemic stroke patients, reported mean ONSD values have ranged between 4.79 mm and 5.85 mm [11-13,15]. Notably, Batur et al. identified a cut-off value of 5.05 mm (sensitivity=96.8%; specificity=95.6%) for detecting increased ICP in ischemic stroke patients [13]. In our cohort, the mean ONSD was 4.86±0.92 mm, which lies close to the upper range of reported normal values but below the thresholds most consistently associated with raised ICP. There are two potential explanations for our finding of a mean ONSD within the normal-to-borderline range compared with previous reports. First, based on the mean NIHSS score, our sample predominantly represented patients with mild stroke severity, in whom a marked rise in ICP would not be expected. Second, cerebral edema typically peaks 3-5 days after stroke onset and is generally not apparent within the first 24 hours, except in cases of large cerebellar infarctions [23,24]. Given that the median time from symptom onset to ED presentation in our cohort was 14 hours, this interval may have been insufficient for a significant increase in ONSD to develop.

The study's limitations include its single-center design and relatively small sample size, with most patients presenting with low NIHSS scores and small infarction areas, which may limit generalizability. In addition, ONSD measurement is operator-dependent. Although formal inter- and intra-observer reliability analyses were not performed, all measurements were conducted by experienced operators following a standardized protocol and were independently reviewed. Blinding of assessors to clinical data was applied to minimize bias. In addition, the study lacked a comparative healthy control group, which could have strengthened the interpretation of ONSD changes. Future studies with larger cohorts could include formal reliability assessments to further validate these findings.

## Conclusions

Both right and left ONSD values can be used to assess ONSD with bedside OUS in patients with acute ischemic stroke. Measurement of a single eye is generally sufficient. Increases in ONSD in either eye are positively correlated, and there is no consistent relationship between the side of cerebral ischemia and ONSD values. These findings support the practicality of ONSD ultrasonography as a non-invasive tool in the acute phase of stroke, although the small sample size and predominance of mild stroke cases may limit generalizability.
